# High-Throughput Yeast Aging Analysis for Cryptococcus (HYAAC) microfluidic device streamlines aging studies in *Cryptococcus neoformans*

**DOI:** 10.1038/s42003-019-0504-5

**Published:** 2019-07-10

**Authors:** Erika P. Orner, Pengchao Zhang, Myeong C. Jo, Somanon Bhattacharya, Lidong Qin, Bettina C. Fries

**Affiliations:** 1 Department of Molecular Genetics and Microbiology, Stony Brook University, Stony Brook, NY 11794 USA; 2 Department of Nanomedicine, Houston Methodist Research Institute, Houston, TX 77030 USA; 3 Department of Cell and Developmental Biology, Weill Cornell Medical College, New York, NY 10065 USA; 4 Department of Medicine, Stony Brook University, Stony Brook, NY 11794 USA; 5 Department of Medicine, Northport VA Medical Center, Northport, NY 11794 USA

**Keywords:** Lab-on-a-chip, Fungal pathogenesis, Antimicrobial resistance

## Abstract

*Cryptococcus neoformans (Cn)* is a deadly fungal pathogen responsible for ~ 180,000 deaths per year and despite effective antifungals, treatment failure and resistance to antifungals are increasingly problematic. Aging and age-related phenotypes are prominent virulence traits that contribute to the resilience of *Cn* to host responses and antifungals. Traditional methods to study aging in *Cn* are expensive, inefficient and in need of improvement. Here, we demonstrate the development and use of a High-Throughput Yeast Aging Analysis for *Cryptococcus* (HYAAC) microfluidic device to better study aging and age-associated genes in *Cn*. Compared to traditional methods, the HYAAC is superior in its efficiency to isolate, manipulate and observe old cells for analysis. It allows for the trapping and tracking of individual cells over the course of their lifespan, allowing for more precise measurements of lifespan, tracking of age-related phenotypes with age, and a more high-throughput ability to investigate genes associated with aging.

## Abstract (web summary)

Orner et al. develops a microfluidic device called High-Throughput Yeast Aging Analysis for *Cryptococcus* (HYAAC) that can be used to measure lifespan, cell death, and dynamics of gene expression changes across age. This device may streamline aging studies in *Cryptococcus neoformans* and facilitate investigations in yeast pathogens.

## Introduction

Aging is an important trait conserved in eukaryotes from unicellular yeasts to mammals^1^. Notably, the generational age of pathogenic yeasts, such as *Candida albicans*^2^, *Candida glabrata*^3,4^, *Candida auris*^5^, and *Cryptococcus neoformans*^6–8^, was shown to be closely linked with their resilience in the host environment.

*C. neoformans* grows in the host environment by asexual reproduction, which is characterized by asymmetric divisions of mother cells resulting in phenotypically distinct daughter cells^9^. These asymmetric divisions lead to age-related phenotypes. The total number of budding events before senescence is termed the replicative lifespan (RLS) and median RLS of a yeast population can be altered under stressful conditions like those encountered in the host environment^10^. Generationally aged *C. neoformans* cells exhibit gradual increase in cell size and increased thickness in the cell wall, phenotypic traits that may contribute to the observed increased resistance to antifungals, hydrogen peroxide, phagocytosis, and phagocytic killing^8,9^. Importantly, it was demonstrated in both *C. neoformans* and *C. glabrata* that generationally older cells accumulate during infection, which may contribute to treatment failure^6,11^. With *C. neoformans* infections being responsible for 15% of AIDS-related deaths worldwide^11^, it is prudent to elucidate the role of replicative aging in persistence and treatment failure of this infection.

Replicative aging is predominantly studied employing elutriation, magnetic bead-based labeling and separation, as well as microdissection to separate mother and daughter cells. These assays are time-consuming, inefficient, and costly. With current methods it is not feasible to evaluate large numbers of cells with advanced generational age, which would be, for instance required for studies pertaining to the stochasticity of RLS.

In recent years, microfluidic devices have been developed for aging studies in *Saccharomyces cerevisiae*. Devices are designed to trap and image individual mother cells, growing in continuously flowing media, which washes away daughter cells from their mothers and provides nutrients required for growth (see review^12^ for a comprehensive description of devices). Devices are bonded to a glass slide so that each cell division can be recorded and scored by time-lapse imaging on a microscope. Available devices, however, were all designed for *S. cerevisiae* and not *C. neoformans. C. neoformans* is a larger yeast that grows in size with increasing age and is surrounded by a polysaccharide capsule, which contributes to cells clumping or sticking together^13^. In devices made for *S. cerevisiae*, *C. neoformans* either outgrew the isolation buckets and were lost, or, cells stuck to each other and caused clumping and overgrowth within the channel. Due to these characteristics, devices made for *S. cerevisiae* did not work for *C. neoformans* cells. Here, we have designed a new device (to our knowledge) that successfully traps individual *C. neoformans* cells, accommodates the cell size enlargement over generations within the isolation buckets, and substantially decreases the likelihood of cells sticking and clumping within the channel. This device can accurately determine RLS, doubling time, and age-dependent antifungal killing on hundreds of cells. It also provides a platform to visualize how specific genes are upregulated in older cells, which will allow studies with mutants to test what genes are relevant for age-dependent resilience against antifungals.

## Results

### HYAAC device design and setup

**Fig. 1:**
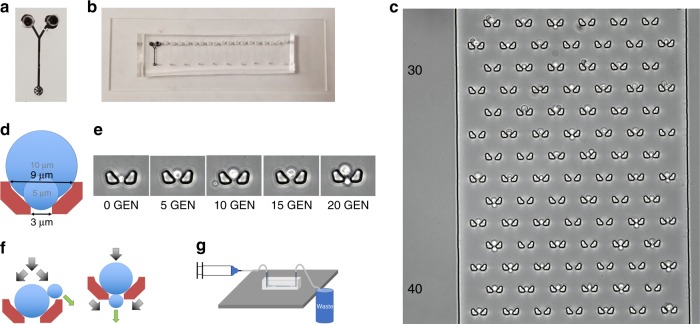
HYAAC chip design. **a** Single HYAAC channel filled with India ink. Channel has dual inlets, 1 cm long channel, and single outlet. **b** Ten HYAAC channels aligned on single, glass microscope slide. **c** Microscope image of cells in buckets arrayed in channel. Each channel has 80 rows of six or seven buckets per row. **d** Bucket designed to hold cells ranging from 5–9+ μm to allow for cell growth over generational aging. **e** Buckets were designed to allow cells to grow in size with age without growing out of the bucket and getting washed away. **f** Diagram of bud removal from mother cells. Black arrows represent media flow direction, green arrows represent the direction of bud movement after separation. **g** Diagram of device setup. Syringe containing media pumps through tubing into the inlet of the device. Tubing is also connected to the outlet, which allows media to flow from the outlet into a waste container. Device is setup on inverted microscope stage in order to have images captured over time

Our High-throughput Yeast Aging Analysis for *Cryptococcus* (HYAAC) device was based on 2 designs originally created for *S. cerevisiae*^14,15^. This device was modified to contain a single channel with two inlets and a single outlet (Fig. 1a, Supplementary Fig. 1). Flow must be continuous in the device to ensure cells remain trapped and thus the volume that can be pushed through the device was limited to the volume a syringe can hold. In order to increase this volume and thus the length of an experiment, a second inlet was added to allow the connection of two syringes at the same time to permit the exchange of syringes without flow interruption. Doubling the perfusion volume permitted the prolongation of experiments in the device and even more importantly, two inlets allowed switching between two different media conditions. To increase the number of strains or media conditions that could be analyzed simultaneously, ten individual channels were aligned on a single microscope slide (Fig. 1b). Within each channel are 80 rows, alternating between six or seven isolation buckets in each row (Fig. 1c). The design of the buckets was modified from their original shape (Supplementary Fig. 2), which was narrow with straight walls. As *C. neoformans* cells grew with age, they outgrew the buckets and escaped through the top of the bucket once they were too large to fit in the width of the bucket. This caused the loss of a number of cells, which interfered with our ability to monitor cells over the course of their lifespan. The new walls were designed to be angled where the bottom of the trap is narrow (3 µm wide), but the top of the trap is wider (9 µm). This is intended to trap cells as small as 4 µm in diameter and allow these cells to grow to at least 10 µm as they generationally age (Fig. 1d, e). The height of the channel was fabricated to be between 10–12 µm to ensure cells would not get stuck between the floor and ceiling of the device as they age. Importantly, this design allows trapped cells to bud in a variety of ways to better ensure daughter cells are washed away. Trapped cells tended to roll within the bucket due to the constant flow of media over the cells (Supplementary Movie 1) until buds begin to form. The bud either got caught at the top of the bucket where it was washed away, or it got trapped in the outlet of the bucket and was washed away (Fig. 1f). In either case, bucket shape was designed to properly allow each bud to be washed away from the mother cell and not clump or cause the mother cell to be washed away (Supplementary Movie 2). Approximately 62% of cells were retained for the entire lifespan in the device. The majority of cells lost are washed out in the first seven generations (Supplementary Fig. 3).

Using stainless steel pins, the device was connected to tubing attached to one or two syringes filled with media (depending on the volume needed for an experiment). Each syringe was placed in a syringe pump which was set to flow at a constant speed of 10–15 µL min^−1^. Another tube was connected to the outlet which flowed into a waste bucket (Fig. 1g). The HYAAC device sat atop a microscope stage on an inverted microscope, which allowed images to be taken over time.

### Replication of *C. neoformans*

**Fig. 2:**
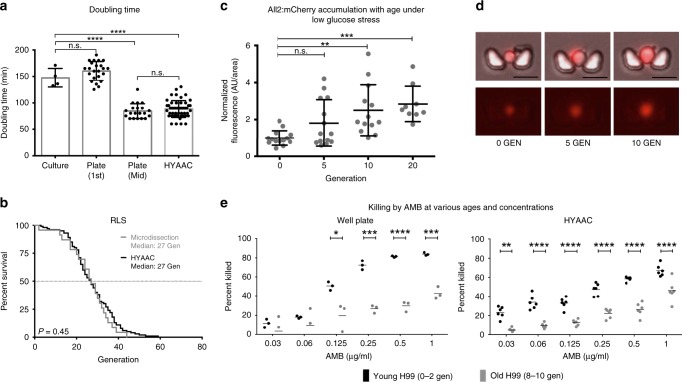
Validation and new uses for the HYAAC chip. **a** Doubling time measured on YPD plate during first replication or mid-lifespan, in YPD culture, or in YPD in HYAAC chip. Error bars represent standard deviation (s.d.). Significance measured by one-way ANOVA and Tukey’s multiple comparison test (*n* *=* 4 independent culture replicates, *n* *=* 50 cells in HYAAC, or *n* *=* 20 cells on plates, df = 37) Box represents mean and error bars represent s.d. **b** Validation of replicative lifespan (RLS) measurement compared to traditional microdissection technique. *P* = 0.450 by Wilcoxon rank-sum test (*n* = 100 cells on HYAAC, *n* = 30 cells by microdissection). **c** Average relative fluorescence **(**normalized to 0-Generation cells [AU/area]**)** of cells with mCherry tagged All2p normalized to cell area then normalized to 0-Generation cells. Error bars, s.d. Significance measured by one-way ANOVA and Dunnett’s multiple comparison test (*n* *=* 14 cells, df = 46). **d** Representative images of an individual cell with mCherry tagged All2p as it grows in age. Scale bars = 10 μm. **e** Validation that HYAAC chip can reproduce trend seen in well-plate antifungal killing assay where old cells are killed less frequently than young cells at various concentrations of Amphotericin B (AMB). Error bars represent mean. Significance measured by student’s *t*-test. *P *<* 0.05, **P *<* 0.01, ***P *<* 0.001, ****P *<* 0.0001

Doubling time was compared for cells grown on YPD plates, in YPD broth, and in the HYAAC device (Fig. 2a). Only mother cells (cells that divided at least once within the device) were analyzed in the HYAAC device as time before entering the traps could not be accounted for. There was no statistical difference between the doubling rates of cells in the HYAAC device (88.1 min, *n* *=* 50 cells) or cells on a YPD plate that were mid-lifespan (84.3 min, *n* *=* 20 cells) (*P* *=* 0.80). Furthermore, no statistically significant difference was noted between the doubling rates of naïve daughter cells grown on YPD plates at 37 °C (1st budding event) (160 min, *n* = 20 cells) or an exponential culture of cells in 50 mL of YPD broth shaking at 150 rpm at 37 °C (147.3 min, *n* = 4 biological replicates) (*P* = 0.46). Mother cells in the HYAAC device, however, grew significantly faster than naïve daughter cells on a YPD plate (*P* < 0.001) and faster than cells in liquid culture (*P* < 0.0001).

### Replicative lifespan of *C. neoformans*

Using this modified HYAAC device, previous findings obtained by traditional methods were validated and experiments were designed to evaluate aging characteristics. Traditionally RLS of *C. neoformans* is measured by streaking a colony onto a YPD plate and manually dissecting and counting each division of 20–30 cells using a dissection microscope and a fiberoptic needle connected to a micromanipulator^6^. The RLS of the wild-type strain H99 has been validated and no statistical difference between cells grown in YPD in the HYAAC device compared to cell dissected by traditional methods on YPD agar was observed (*P* = 0.450) (Fig. 2b, Supplementary Fig. 4). Approximately 100 cells were followed in the HYAAC for their full lifespan and compared to 23 cells analyzed by traditional microdissection. Only cells retained through their entire lifespan were analyzed (Supplementary Fig. 3). The RLS, when dissected on a plate, ranged from 2 to 44 generations with a median of 27 generations (*n* = 23). The RLS in the HYAAC ranged from 3 to 59 generations with a median of 27 generations (*n* = 100). Though there was no statistical difference between the HYAAC device and traditional dissection, specific differences in the longest living cells were noted. In the HYAAC device, the cells that lived more than 1 standard deviation (9.7 generations) above the median RLS (27 generations) lived between 38 and 59 generations whereas the cells that lived more than 1 standard deviation (10.4 generations) above the median RLS (27 generations) of cells that were microdissected only lived between 33–44 generations (Supplementary Fig. 4).

### Protein accumulation with age under stress

The HYAAC device can be used to quantitate protein levels within individual cells at different generational ages of a population. Here, levels of the allergen 2 protein (All2p) labeled with mCherry were quantified using the HYAAC device (Fig. 2c, d). All2p is a protein that is responsible for maintaining intracellular pH in low-pH conditions in *C*. *neoformans* and is upregulated when subjected to low glucose conditions. Furthermore, deletion mutants exhibit shortened RLS under low glucose conditions^16^. Given that older *C. neoformans* cells are more resistant to H_2_O_2_^8^ and given the role of All2p in pH homeostasis, we hypothesized the protein would accumulate during aging in low glucose conditions. Using the HYAAC chip, relative protein levels were compared among various generational ages. Bright field and fluorescent images of cells were captured as individual cells grew from 0 to 5 to 10 to 15 to 20 generations under no stress or under the stress of caloric restriction (0.05% glucose). Relative protein levels were quantified from fluorescence measured in each tracked cell using ImageJ software as each cell aged. Cells grown in normal glucose conditions did not fluoresce, indicating there was no protein accumulation. Fluorescence from cells grown under low glucose conditions, however, were normalized first to cell size and then to 0-generation cells. Mean fluorescence for 5-generation-old cells was 1.81 (*P* *=* 0.1271), mean fluorescence for 10-generation-old cells was 2.50 (*P* *=* 0.0019), and mean fluorescence for 20-generation-old cells was 2.84 (*P* *=* 0.0006). We observed that not only did the All2p levels rise under low caloric stress, but All2p accumulated to even higher levels in individual cells as they aged to older generations. Furthermore, we noted that there was large variability of fluorescence between individual cells of similar generational age (as noted by large standard deviation bars).

### Resilience of older cells to antifungal killing

Of great importance for pathogenic yeasts is their resilience to antifungal agents and how age-dependent resilience contributes to the persistence of old cells during infection^6^. The HYAAC device was used to evaluate age-dependent resilience to antifungals as this attribute is not readily measured by standard laboratory methods (Fig. 2e). For standard killing assays, newly budded yeast cells were biotin labeled and grown to a desired generational age. As surface biotin is not transferred to daughter cells during division, original biotin-labeled yeast cells of a specific age were then separated from their unlabeled daughters by magnetic bead sorting via magnetically-conjugated streptavidin. These original, generationally older cells (9–11 generations) were then used and compared to the non-labelled fraction comprised mostly of a young (1–3 generations) population of cells in an antifungal killing assay. The non-labelled fraction was used as a control as they were exposed to the same manipulations as the old fraction. Both old and young cells were exposed in a 96-well plate to various concentrations of Amphotericin B for 3 h and plated on YPD with no drug. Amphotericin B was chosen as it is the standard treatment for cryptococcosis. The number of surviving colony forming units were normalized to cells that were not subjected to antifungals. These findings demonstrate that older cells are killed significantly less compared to young cells at 0.125 µg mL^−1^, 0.25 µg mL^−1^, 0.5 µg mL^−1^, and 1 µg mL^−1^ of Amphotericin B (50.6% young vs. 19.7% old cells killed, *P* *=* 0.025, 72.2% young vs. 26.9% old cells killed, *P* *<* 0.001, 81.4% young vs. 29.8% old cells killed, *P* *<* 0.0001, and 84.0% young vs. 43.1% old cells killed, *P* *<* 0.0001, respectively) when using the traditional well-plate methodology. These findings are consistent with previously shown findings of killing assays with various antifungals^6,8^.

Next, resilience of old cells was examined using the HYAAC device. Specifically, trapped cells were aged for 15 h in the HYAAC device in the absence of antifungals to an average of 10 ± 2 generations. Media with a range of concentrations of Amphotericin B was then flowed through the channel for 3 h, analogous to the exposure times in the well-based killing assay. After 3 h, media with no antifungals were reintroduced and the number of cells that continued to divide and the number of cells that did not divide as a result of the antifungals were counted and compared. Using this HYAAC-based technique, 8–10-generation-old *C. neoformans* cells exhibit higher resilience to killing by Amphotericin B over a range of concentrations when compared to 0–2 generation old *C. neoformans* cells. Again, older cells were killed significantly less than young cells at 0.03 µg mL^−1^, 0.06 µg mL^−1^, 0.125 µg mL^−1^, 0.25 µg mL^−1^, 0.5 µg mL^−1^, and 1 µg mL^−1^ of AMB (23.3% young vs. 5.01% old cells killed, 33.8% young vs. 9.5% old cells killed, 33.0% young vs. 12.7% old cells killed, 47.3% young vs. 22.3% old cells killed, 58.5% young vs. 26.3% old cells killed, and 67.2% young vs. 46.0% old cells killed, respectively. *P* *<* 0.01 for all). Importantly, the HYCAA device could document difference in resilience between young and old cells at Amphotericin B concentrations as low as (0.03 µg mL^−1–1^ µg mL^−1^), whereas the well-based assay only showed statistical differences at Amphotericin B concentration down to 0.125 µg mL^−1^.

### Genes upregulated in aging contribute to resilience

**Fig. 3:**
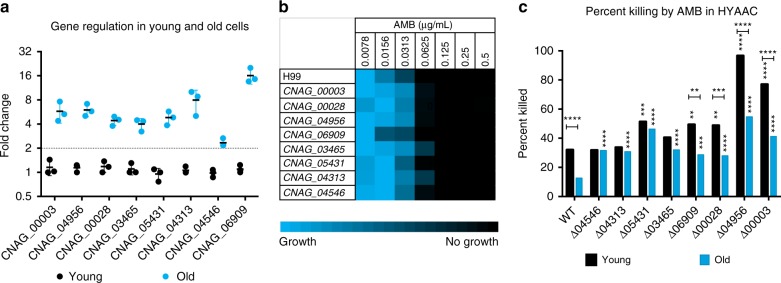
Age-associated gene characteristics. **a** Fold change of ten genes in young and old wild-type cells found by qRT-PCR analysis normalized to β-actin. Middle bar represent mean, error bars represent s.d. Fold change above 2 (dotted line) was considered significant. *n* *=* 3 biological replicates. (**b**) MIC analysis of wild-type and knockout strains. Brighter blue indicates more growth while black indicates no growth. *n* *=* 3 biological replicates. **c** Percent of old and young cells killed in the HYAAC device when subjected to 3 h of 0.125 μg mL^−1^ Amphotericin B for wild-type and all knockout strains. *n* = 200 cells per strain. Error bars represent s.d. Significance measured by Chi-square analysis. Horizontal *P *<* 0.05, **P *<* 0.01, ***P *<* 0.001, ****P *<* 0.0001 comparisons done between young and old of same strain. Vertical *P *<* 0.05, **P *<* 0.01, ***P *<* 0.001, ****P *<* 0.0001 comparisons done between wild-type and mutant

To further explore what genes may be contributing to the increased resilience of older cells, we chose to investigate eight genes that are differentially expressed in the process of aging (Table 1). These genes included two high-affinity nicotinic acid transporters (*CNAG_00028* and *CNAG_04956*) and NADPH dehydrogenase (*CNAG_04313)* as previous work has shown that drugs that target nicotinic acid affect RLS^17^, three drug transporters (*CNAG_00003*, *CNAG_04546*, and *CNAG_06909)*, and two cell wall-associated genes that have been shown to contribute to the resilience of *C*. *neoformans*, *Lac1*^18^, and *Rim101*^19^ *(CNAG_03465* and *CNAG_05431*, respectively*)*. To assess whether transcription of these genes was regulated during replicative aging, qRT-PCR on 0-generation-and 10-generation-old cells was performed. All genes exhibited more than 2-fold upregulation in older *C*. *neoformans* cells (Fig. 3a) compared to young cells.

**Table 1:** Upregulation of genes in 10-Generation cells compared to 0-Generation cells

Gene	Gene name/function	qPCR fold change
*CNAG_00003*	Drug transporter	5.77
*CNAG_04956*	High-affinity nicotinic acid transporter	5.99
*CNAG_00028*	High-affinity nicotinic acid transporter	4.42
*CNAG_06909*	ABC transporter family protein	16.06
*CNAG_05431*	*Rim101*, pH response transcription factor	4.81
*CNAG_03465*	*Laccase 1*, melanin biosynthesis	4.00
*CNAG_04313*	NADPH dehydrogenase 2	7.97
*CNAG_04546*	Multidrug transporter	2.34

In order to determine whether these genes are contributing to the increased antifungal resilience of older cells, mutants were aged and subjected to AMB killing in the HYAAC device. Antifungal resilience between old and young mutant cells was compared to that of young and old wild-type cells. Standard MIC testing demonstrated that minimum inhibitory concentration (MIC_80_) for AMB was 0.0625 µg mL^−1^ for all eight mutants, unchanged from that of wildtype (Fig. 3b).

Next, the HYAAC device was used to test if the age-dependent AMB resilience was altered in mutants. Both young and old cells of mutants were subjected to 0.125 μg mL^−1^AMB for 3 h in the HYAAC device. This was the lowest concentration where >50% of death was observed in the killing assay done on wild-type cells. At least 200 cells per strain were assessed on the HYAAC and percent of cells killed after antifungal exposure was calculated and data were compared by Chi-square analysis (Fig. 3c, Table 2). Overall, old cells of all mutants are significantly more susceptible to Amphotericin B in the HYAAC device when compared to wildtype (*P* *<* 0.001). Similarly, young cells of five of the eight mutants (*∆CNAG_00003, ∆CNAG_04956, ∆CNAG_00028, ∆CNAG_06909*, and *∆CNAG_005431*) were also significantly more susceptible to 3 h of 0.125 μg mL^−1^ Amphotericin B (*P* *<* 0.01). Most importantly, analysis on the HYCAA device identified four mutant strains that showed no significant difference in killing between young and old cells (*∆CNAG_04546, ∆CNAG_04313, ∆CNAG_05431*, and *∆CNAG_03465, P* *=* 0.9789, 0.5791, 0.4117, and 0.1214, respectively*)*. The lack of age-acquired resilience was most notable in *∆CNAG_04546* and *∆CNAG_04313* as these two mutants exhibited unchanged baseline killing compared to wildtype (about 32%) with no increase in resilience when aged to ten generations. This indicates these genes may be contributing to the age-dependent resilience to Amphotericin B seen in wild-type cells.

**Table 2:** Age-dependent killing of cells after 3 h of 0.125 μg mL^−1^ Amphotericin B treatment

Strain	10-generation percent killed	0-generation percent killed	0 v 10-generation *P* value	Fold change	Percent change
WT	12.7	32.0	<0.0001	2.52	60%
*∆CNAG_00003*	41.3^****^	76.9^****^	<0.0001	1.86	46%
*∆CNAG_04956*	54.7^****^	96.5^****^	<0.0001	1.76	43%
*∆CNAG_00028*	28.0^****^	48.7^**^	0.0001	1.74	43%
*∆CNAG_06909*	28.7^***^	49.3^**^	0.0012	1.72	42%
*∆CNAG_03465*	32.1^****^	40.5	0.1214	1.26	21%
*∆CNAG_05431*	46.3^****^	51.3^***^	0.4117	1.11	10%
*∆CNAG_04313*	30.8^****^	33.7	0.5791	1.09	9%
*∆CNAG_04546*	31.7^****^	31.8	0.9789	1.00	0%

**P *<* 0.01, ***P *<* 0.001, ****P *<* 0.00001 by Chi-square analysis compared to wildtype of the same column. *n* *>* 200 cells for all strains.

## Discussion

Most research on *C. neoformans* pathogenesis is performed with exponential cultures, which are predominantly made up of naïve and young cells. Our previous work suggests that during infection, older *C. neoformans* cells are selected and persist while younger cells are preferentially killed^6^. A similar selection of old cells has also been documented in *Candida glabrata* infections^4^. Given that aging is a regulated process that could potentially be targeted for antifungal drug development, we sought to develop better methods to study this unique mechanism of age-related resilience. Current techniques to isolate old cells are time-consuming, expensive, inefficient, unreliable, and laborious. To combat these issues, we have developed a microfluidic HYAAC platform that allows us to not only isolate single, aged cells, but also subject them to a number of clinically relevant conditions and evaluate the response of generationally older cells to selective pressures on an individual cellular level.

Here we have demonstrated several major advantages of the HYAAC device. Foremost, this methodology not only improves time efficiency, but it also improves cell separation efficiency. Few cells are lost over time in the device but the same cannot be said using traditional magnetic separation, which has a cell retention rate ranging from 20 to 90% depending on the strain and the number of separations they go through. Here, although some cells are lost over time in the device (Supplementary Fig. 3), the HYAAC device still has a retention rate near 62% and most cells that are lost are lost within the first seven generations. Furthermore, we can track hundreds of individual cells over the course of their replicative lifespans while subjecting them to various growth conditions. Although this system will expedite and quasi-automate the microdissection procedure, it is not a true high-throughput system as it can only manage a limited number of strains simultaneously. In addition, there is still no good software to analyze all the data reliably. Hence it would still be very time and labor intensive to study a complete mutant collection. However, this device greatly reduces experimental time required to obtain results. A lifespan experiment can be performed without interruption overnight as is necessary during microscope dissections cutting the duration of an experiment down from weeks to days. This also increases accuracy of RLS as mother cells are not subjected to the stresses of constant temperature changes or physical stress of manual manipulation. Such stress factors may not affect the median lifespan of a yeast population but may interfere with analysis of variance and stochasticity which focus on RLS of outliers, specifically the longest living cells. Indeed, our lifespan analysis from the HYAAC device documented cells that can live longer in the HYAAC device compared cells analyzed by standard dissection methods. Alternatively, the noted discrepancy in RLS on long-living outliers also could be due to the fact that lifespan determined by standard methods is obtained on a relatively small number of cells. Hence, the HYAAC system is superior as it greatly increases the number of individual cells that are assessed, which permits modeling of stochasticity and variance within a population.

HYAAC-derived data analysis indicated that mother cells exhibit a shorter doubling time than the average doubling time derived from the same yeast strain grown in vitro. This finding is consistent with data from *S. cerevisiae* in that naïve daughter cells take longer to divide than mother cells who have divided at least once^20^. The doubling rate of an exponential culture reflects that of a mixed population, composed of both mother and daughter cells and thus the doubling rates is an average of doubling rates of mother and daughter cells.

Another potential application of the HYAAC device is that this method allows assessment of protein expression and individual cell phenotypes as a function of generational age. We were able to visualize and quantify protein levels or gene expression in an individual mother cell as it ages within the device. All2p is a protein that regulates pH homeostasis and *C. neoformans* has a decreased RLS when this gene is knocked out, indicating that it may be critical for longevity. We hypothesized that All2p would not only accumulate under low glucose stress, but also accumulate in generationally older cells. As expected, All2 protein levels increased with generational age when grown in low glucose conditions. We also noted there was large variability of protein expression within individual cells, especially as they age. Few investigations have aimed to understand the stochasticity of RLS. Previous papers have reported a wide range of RLS of individual cells within a population^21–25^. One intriguing hypothesis, which can now be examined, is the possibility that cells with extreme lifespans exhibit altered protein expressions allowing them to persist in harsh environments. RLS analysis of a collection of clinical strains in three different yeast species, including *C. neoformans*^6^, *C. glabrata*^4^, and *C. auris*^5^, has revealed remarkable variability of RLS among individual cells within a single strain as well as among different strains. It is now feasible with the HYAAC device to measure protein expression on a cellular level and correlate it with the generational age of individual cells within a population. In that regard, recent experiments that involved modeling of large transcript and protein expression data sets in *S. cerevisiae* indicate that targeting protein synthesis or downstream consequences may intervene in aging^26^. Findings from such data sets can be used to do targeted investigations regarding the relevance of pathways that could alter the process of aging and resilience.

Of particular importance, the HYAAC device can be used to assess variability in resilience to antifungals in individual yeast cells. Notably enhanced sensitivity to lower concentrations of Amphotericin B was documented in the HYAAC device compared to a well-plate assay. This may occur because cells in the HYAAC device are exposed as individual cells as opposed to clumps of cells seen in suspensions or when cells sediment to the bottom of a well. This singularity may prevent shielding of yeast cells from antifungals. Indeed, mathematical models have suggested there is a tradeoff between unicellularity and multicellularity of yeast cells, especially under stress. It has been shown that while multicellular clumps of cells are more resistant to stressors like temperature changes, ethanol, and hydrogen peroxide, unicellular yeasts are more fit under no stress^27^. Since clumping and yeast aggregation is abundant in cryptococcomas, it would be of interest to study the resistance of small clumps (2–6 cells) on the HYAAC device as long as they still permit flow and compare differences in antifungal resilience between unicellular and multicellular *Cryptococcus*.

Antifungal resilience is not only important to study on the single cell level, but also with respect to age, as we have previously demonstrated increased age increases antifungal resilience and older cells are the cells that accumulate during infection^6^. The mechanism of age-dependent resilience, however, is not understood. In this study, we began to investigate a number of genes suspected to be involved in age-dependent resilience by assessing age-dependent resilience to Amphotericin B. Mutants were chosen because their deleted gene was upregulated in in the process of aging. We hypothesized that if the gene product was relevant to the observed resilience of old *C. neoformans* cells, then age-dependent increase in resilience would not be observed. These experiments identified four mutants of interest. In *Lac1 (CNAG_03465)*, *Rim101 (CNAG_05431)*, *NADPH dehydrogenase II (CNAG_04313)*, and multidrug transporter (*CNAG_04546*) knockout mutants, we found that there was no longer a significant difference in killing by Amphotericin B between young and old cells when compared to wild-type cells (*P* *=* 0.1214, 0.4117, 0.5791, and 0.9789, respectively). The multidrug transporter *CNAG_04546, Lac1*, and NADPH Dehydrogenase II are genes that are of particular interest to further study as these knockout mutants showed no significant change in overall susceptibility to Amphotericin B killing (*P* *=* 1, 0.0917, and 0.7551, respectively) and old cells were killed at the same rates as young cells. This indicates upregulation of these genes is likely contributing to the resilience of old cells to Amphotericin B. *Rim101* is also of interest despite cells showing slightly increased susceptibility overall, as there was no significant difference in killing of old and young cells of the knockout mutant strain (*P* *=* 0.4117*)*. The other four mutants, *CNAG_00003, CNAG_04956, CNAG_00028*, and *CNAG_06909* still showed significant differences in killing between young and old cells (*P* ≤ 0.0012*)*. However, they were overall more susceptible to 3 h of 0.125 μg mL^−1^ Amphotericin B treatment compared to wildtype so these genes may be slightly contributing overall resilience to Amphotericin B, but as the MICS are not different, they are not likely playing a large role.

Despite major improvement, some challenges with the device still remain. Prolonged RLS assays continue to have a higher risk of failure because the device is more likely to clog and over grow in prolonged runs. Similarly, if the RLS is extremely long, it may not be possible to use syringes of large enough volume to ensure enough media is fed through the device for the entirety of the lifespan. Furthermore, media that induces an increase in capsule size or increase in clumping will be difficult to use as they will increase the likelihood of clumping and overgrowth within the device. Lastly, progress still has to be made with respect to optimizing imaging and data analysis. Software programs that automatically analyze images to calculate generational age and RLS should be developed. Furthermore, microscopes are highly sensitive to any kind of liquid spill so care must be taken to avoid leaks and spills throughout the system. Despite these obstacles, this device will provide a powerful and efficient method to study drug-induced killing assays and experiments that focus on cells aged 0–20 generations. Based on statistical modeling of dividing cell populations, cells that are older than 20 generation are not likely to accumulate in an in vivo expanding population any way^4,6^.

In summary, this method can be used as a valid replacement of outdated, expensive, and difficult techniques to help further study the importance of aging and how it contributes to virulence in not only *C. neoformans*, but other yeast pathogens as well.

## Methods

### Strains and media

All strains and media are listed in Supplementary Tables 1 and 2.

### Traditional isolation of old cells

Methods used to isolate generationally older cells were based on previously described methods^6^. Briefly, naïve cells were labelled with EZ linkTM Sulfo-NHS-LC-LC-Biotin (Thermo Scientific) and then grown in synthetic complete media for aging (SCA) media at 37 °C (Supplementary Table 1) to desired generational age. Cells were then labelled with magnetically-conjugated anti-biotin (Miltenyi Biotec) and filtered and sorted through a magnetic LS column (Miltenyi Biotec). LS columns separate old cells of interest from their young daughter cells which serve as controls, since these cells have encountered the same separation manipulations and stress as their older counterparts.

### Traditional microdissection for RLS

Methods used to measure RLS and doubling time using microdissection were done using previously described methods^6^. Briefly, 25–40 naïve cells were isolated and arrayed on yeast extract, peptone, dextrose (YPD) agar plates. To measure RLS, each time the isolated mother cell budded (every 1–2 h), the daughter cell would be removed and counted. Daughter cells were separated using an Axioscope A1 microscope (Zeiss) outfitted with a 50 µm fiberoptic needle (Cora Styles). After each division, the plate would be returned to a 37 °C incubator to maintain growth conditions. Plates would be stored at 4 °C overnight to slow growth and prevent overgrowth and loss of the mother cells. The RLS was determined by the number of daughters the mother would give off before dying (as defined by no divisions for 1 week).

### Traditional doubling time

Doubling time was calculated in YPD broth and on YPD agar (Supplementary Table 1). On YPD plates, cells were arrayed, and each division was separated by the same methods for RLS determination. Doubling time was determined by measuring the time between each complete budding event (cells were checked every 10 min to be as precise as possible without overly stressing cells or keeping them at room temperature too long). In YPD broth, doubling time was determined using previously published methods^16^. Briefly, overnight cultures were sub-cultured to a starting OD_600_ = 0.2. After a couple hours to allow the cells to start dividing again, OD readings were taken every hour for 6 h at 600 nm using a spectrophotometer. Doubling time was calculated using the formula:

$$T_i \times \frac{{0.693}}{{{\mathrm{ln}}\frac{{{\mathrm{OD}}_{Ti}}}{{{\mathrm{OD}}_{T0}}}}}$$

Where Ti is the number of hours since the first OD reading (OD_Ti_) was taken and OD_To_ is the OD measured at that time. For example, if OD_To_ = 0.21 and 1 h later, OD_Ti_ = 0.287, the doubling time is 2.2 h.

### Traditional antifungal killing

Methods to assess killing by antifungals were done by previously published methods^9^. Briefly, cells were aged to 10 generations in SCA media using the above methods. Ten generation and young control cells were then plated in various concentrations of Amphotericin B in RPMI 1640 (Supplementary Table 1) and were incubated for 3 h at 37 °C. Cells were then diluted, plated on YPD agar plates, and incubated at 37 °C for 2 days, after which colony forming units (CFU) was calculated and percent killing was done by normalizing CFU counts to CFU counts of wells with no antifungals.

### Minimum inhibitory concentration (MIC_80_)

Minimum inhibitory concentration or MIC is defined as the concentration of drug required to inhibit or kill 80% of microbial growth. MICs to amphotericin B was performed as described previously^28^. Amphotericin B was first added in flat-bottomed 96-well plate (Costar) in two-fold serial dilution followed by the addition of the inoculum. The inoculum was prepared as follows. Briefly, *C. neoformans* cells were grown overnight in SAB media (Difco). The overnight cultures were washed three times with PBS and 0.1 OD_600_ of cells were used as inoculum. In the 96-well plate, one row with only cells and no drug was used as a growth control, while one row with only media and no cells were used as contamination control. Absorbance readings were taken at OD_600_ at 48 h (SpectraMax i3X). The assay was done in triplicate and the average MIC values are reported.

### RNA isolation and quantitative reverse transcriptase PCR

RNA from young (0–3 generation) and old (10 generation) cells were isolated using RNAeasy kit (Qiagen) following manufacturer’s recommendation. The isolated RNA was quantified using nanodrop BioSpectometer (Eppendorf), and absorbance ratios (A260/A280) of ≥2.0 were considered as pure RNA. Two hundred nanogram of RNA from both young and old cells was then converted to cDNA using Verso cDNA synthesis kit (Thermo Scientific) following manufacturer’s guidelines. qPCR on the cDNA was done using Power Sybr Green (Applied Biosystems) and the oligonucleotides listed in Table S3 to quantify gene expression in young and old. Gene expression was normalized to the house-keeping gene *ACT1* and was calculated by using 2 ^-⊗⊗Ct^ method as described previously^29^. qPCR assay was done in triplicate, and gene expression above two-fold was considered to be significant. Primer sequences are detailed in Supplementary Table 3.

### HYAAC device design

The HYAAC chip was based on previous designs^14,15^ but was modified to have a single channel with inlets and a single outlet. Each channel is comprised of 80 rows of staggered buckets that alternate between six or seven buckets per row. The buckets were designed to be modified triangles facing each other where the bottom of the bucket is 3 µm wide and the top of the bucket is 9 µm wide. This allows for the cell growth that occurs with aging. Ten HYAAC channels are lined up on a single microscope slide.

### HYAAC device fabrication

The HYAAC device was designed using AutoCAD software, printed out as 5-inch glass photomasks (Photo Sciences Inc.), and fabricated using soft lithography with polydimethylsiloxane (PDMS) molding technique^5^. Briefly, a spin-coat of photoresist (SU-8 3010, MicroChem Corp.) was spun onto a 5-inch silicon wafer (Silicon Quest International Inc.) at 3000 rpm for 30 s (Laurell Technologies Corp.). The spin-coated wafer was then soft-baked at 65 °C for 3 min followed by a hard-bake at 95 °C for 10 min. The device design was patterned onto the wafer by exposure to ultraviolet (UV) light at an exposure dose of 150 mJ/cm2 for 3 s through the photomask. After baking at 95 °C for 3 min and developing in the SU-8 developer for 3 min, the wafer was exposed to UV light for another 20 s and hard-baked at 135 °C for 30 min. The master was then silanized with trimethychlorosilane for 30 min to make the surface hydrophobic and assist in replicate casting. Each device was cast using PDMS (10 A:1B; Sylgard 184 kit from Dow Corning Corp.), which was first degassed in a vacuum and allowed to cure atop the master mold at 80 °C for 30 min. Each PDMS device was peeled off the master mold and inlets and outlets were punched through the device using a biopsy punch (0.5 mm Inner Diameter; Harris Uni-Core). The PDMS device was irreversibly bonded to glass slide after treating the surfaces of both the device and glass slide with oxygen plasma (1 min at 20 sccm oxygen flow rate, 500 mTorr chamber pressure, and 50 W power) and aligning the device atop the slide.

### HYAAC setup and utilization

Cells were grown overnight in SCA media and subculture for 6 h the next day to ensure a young, exponentially growing culture. Cells are then diluted and loaded into the HYAAC using a 1 mL syringe (BD) connected to Tygon microbore tubing (Cole-Parmer). The HYAAC chip is then set atop an onstage incubator (Tokai) of an inverted microscope that keeps the chip and its contents at 37 °C. Larger syringes were filled with the media of choice and loaded into a syringe pump (KD Scientific), which pumps the media through the HYAAC channel and a constant rate of 10–15 µL min^−1^. This constant flow of media provides fresh media to the cells and keeps them trapped in the buckets so individual cells can be monitored for the entirety of the experiment.

RLS in the HYAAC device is measured by growing cells in YPD in the HYAAC and capturing each budding event through microscope imaging and counting each division before the cells dies. Doubling time is measured by capturing images every 5 min and calculating the time it takes for the mother cell to complete each division. Antifungal susceptibility testing was done by growing cells in the HYAAC in SCA media until they reached 10 generations and then flowing Amphotericin B in SCA over the cells for 3 h before switching back to SCA media without Amphotericin B and counting how many cells continue to divide or stop dividing.

### Image acquisition and analysis

Time-lapse images were taken every 5 or 20 min using the EVOS® FL Auto Imaging System equipped with an automated stage to allow for multiple X, Y positions to be imaged at each time point. Images were taken using a ×40 objective lens. Images acquired were analyzed by the open-source image processing software, FIJI in order to assess RLS, doubling time, and antifungal susceptibility and to quantify fluorescence.

### Statistics and reproducibility

Significance was evaluated using a Wilcoxon rank-sum test for RLS measurements or a two-tailed student’s *t*-test. Doubling times were compared using a one-way ANOVA and Tukey’s multiple comparison post-hoc test. mCherry fluorescence quantification was compared using a one-way ANOVA and Dunnett’s multiple comparison post-hoc test. Differences between young and old cell killing by antifungals in a well plate were compared using a two-tailed student’s *t*-test. Percent killing in the HYAAC was compared using Chi-square analysis comparing young and old cells that survived or died and comparing young wild-type cells to young mutant cells and old wild-type cells to old mutant cells. All experiments were done in biological triplicates unless otherwise noted.

### Reporting summary

Further information on research design is available in the Nature Research Reporting Summary linked to this article.

## Supplementary information

Supplementary Information

Description of Additional Supplementary Files

Supplementary Data 1

Reporting Summary

Supplementary Movie 1

Supplementary Movie 2

## Data Availability

Data generated or analyzed in this study, which are not provided in the Supplementary Data, are available through request from the corresponding author. AutoCAD design of the HYAAC device is available at figshare (10.6084/m9.figshare.8216267). The source data underlying plots were shown in Supplementary Data 1.
