# Supplementary material for: High-Throughput Yeast Aging Analysis for Cryptococcus (HYAAC) microfluidic device streamlines aging studies in Cryptococcus neoformans
Source: Commun Biol. 2019 Jul 10;2:256. doi: 10.1038/s42003-019-0504-5 (PMC6620289; doi:10.1038/s42003-019-0504-5)
Supplement: Supplementary file 1 — Supplementary Information [file 42003_2019_504_MOESM1_ESM.pdf]

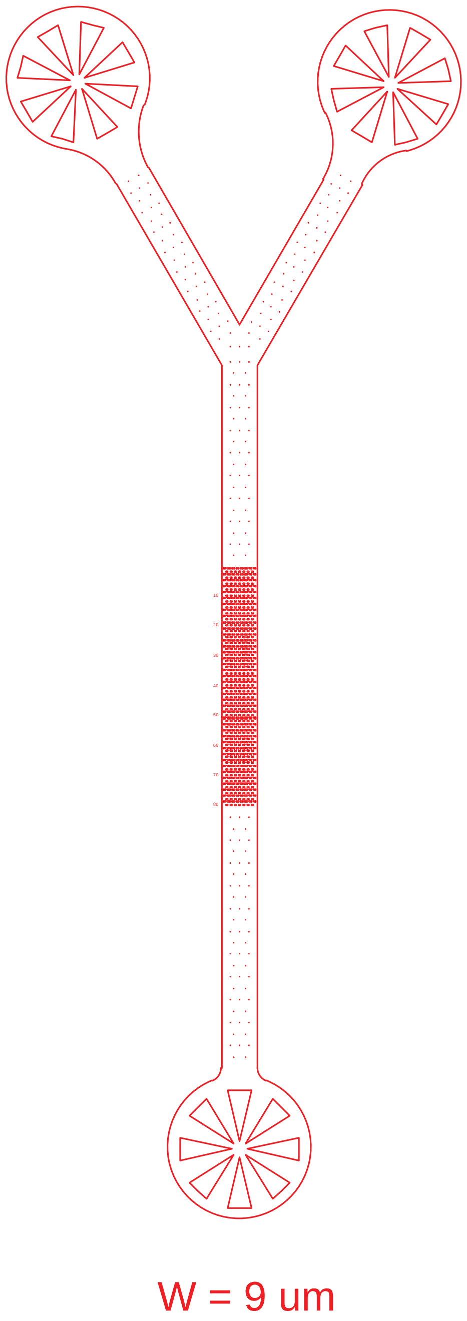


**Supplementary Figure 1:** Template outline of one HYAAC channel as designed in AutoCAD.

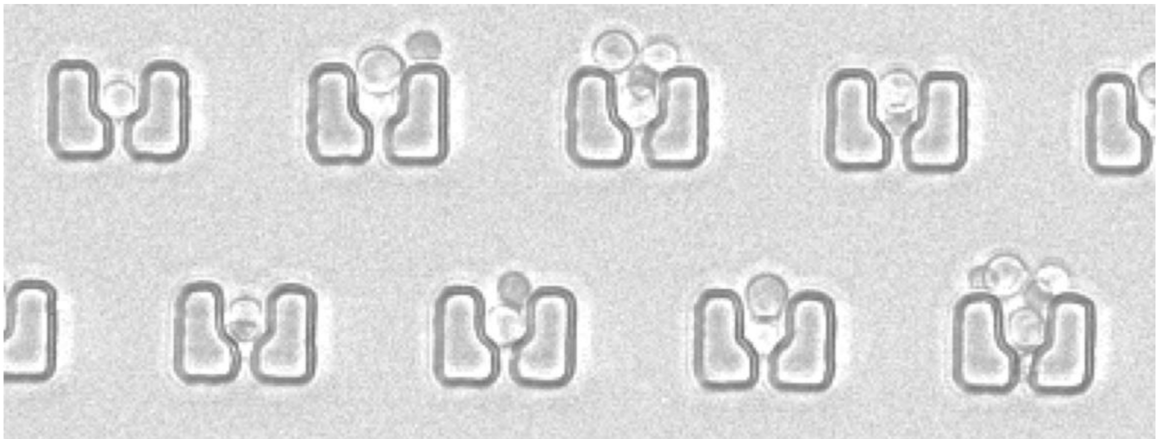

**Supplementary Figure 2:** Original buckets of HYAA chip filled with *C. neoformans*. Numerous issues were found with the original buckets where cells could outgrow through the top or clump and cluster together.

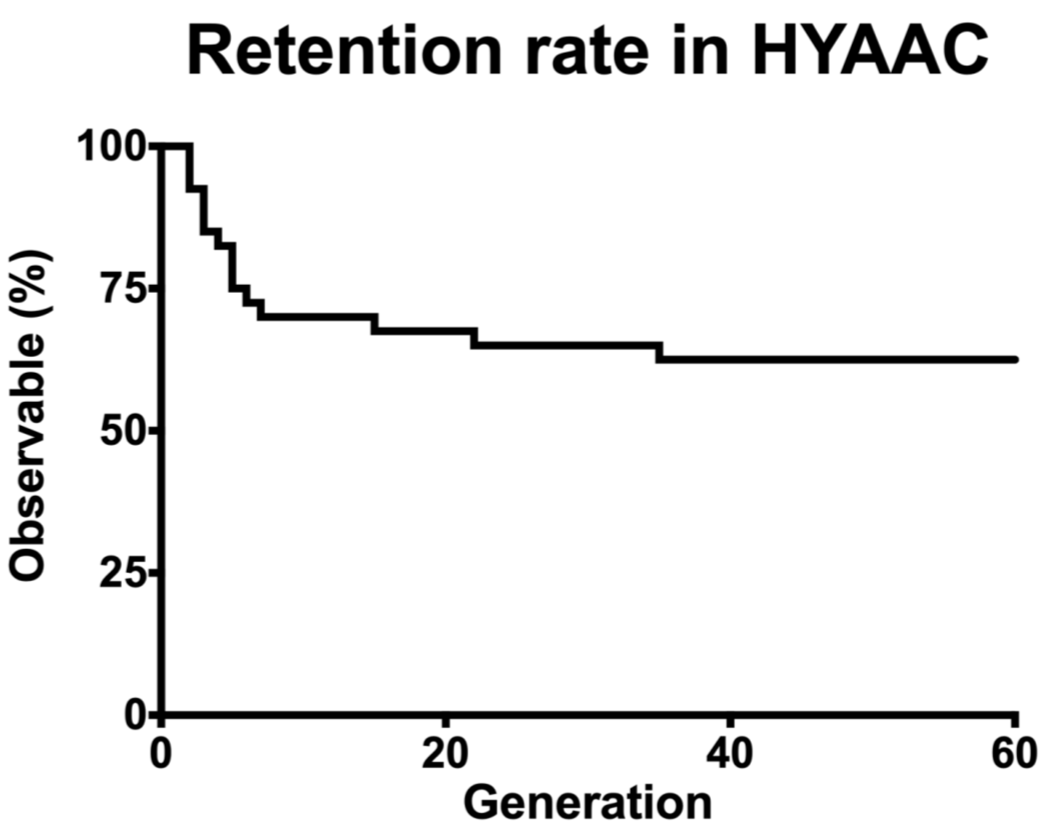


**Supplementary Figure 3:** Retention rate for cells that landed in a trap and replicated at least once. 28% of cells were lost within the first 7 generations and there was an overall retention rate of 62% for the 50 wildtype cells assessed.

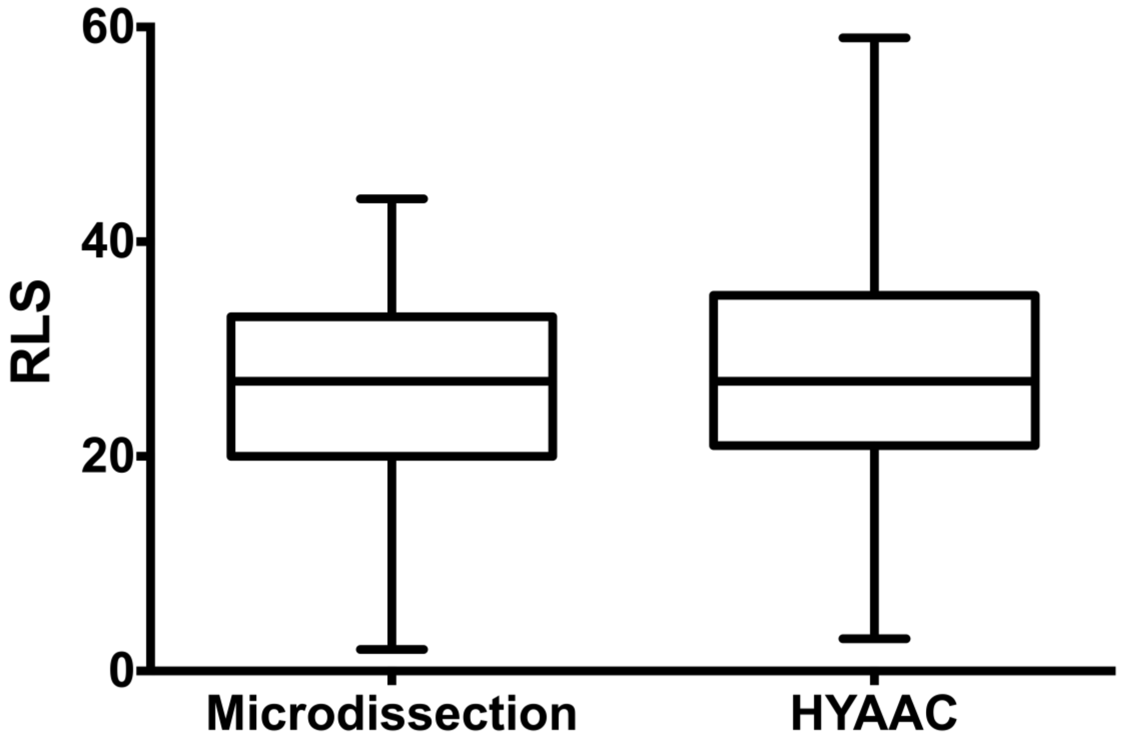


**Supplementary Figure 4**: RLS of WT cells as measured by microdissecion ($n$ = 30) of in the HYAAC device ($n$ = 100). $P$ = 0.450 by Wilcoxon rank sum test. Center line is median, box limits are upper and lower quartiles, whiskers are minimum and maximum.

**Supplementary Table 1:** All strains used in this paper.

| **Strain** | **Reference** |
|---|---|
| WT | H99 - Provided as a gift from Dr. John Perfect of Duke University. |
| RC2-ALL2:mCherry | Jain et. al, *Infection and Immunity* (2016) |
| H99 Δ*CNAG_05431* | Provided as a gift from Dr. Andrew Alspaugh of Duke University. |
| H99 Δ*CNAG_03465* | Provided as a gift from Dr. Peter Williamson of the National Institute for Health. |
| KN99*α* Δ*CNAG_00003*<br>KN99*α* Δ*CNAG_04956*<br>KN99*α* Δ*CNAG_00028* | Purchased from the Madhani knockout collection managed by the Fungal Genetics Stock Center. |
| KN99*α* Δ*CNAG_04546*<br>KN99*α* Δ*CNAG_04313*<br>KN99*α* Δ*CNAG_06909* | Provided as a gift from Dr. Maurizio Del Poeta at Stony Brook University who purchased from the Madhani knockout collection managed by the Fungal Genetics Stock Center. |

**Supplementary Table 2:** Media used in this paper. Each recipe is adjusted for 1L of media in $dH_2O$.

| **Media** | **Recipe (in 1L $dH_2O$)** |
|---|---|
| YPD broth | 10 g yeast extract, 20 g peptone, 20 g dextrose (Difco) |
| YPD agar | 10 g yeast extract, 20 g peptone, 20 g dextrose, 15 g agar (Difco) |
| Sabourad Dextrose (SAB) broth | 5 g peptic digest of animal tissue, 5 g pancreatic digest of casein, 20 g dextrose (Difco) |
| RPMI 1640 | 10.4 g RPMI 1640 (with L-glutamine, without sodium bicarbonate), 165 ml 1M MOPS, adjusted to pH 7 |
| SCA media | 1.7 g yeast nitrogen base without amino acids, 1 g drop out mix, 0.4% ethanol, 5 g $(NH_4)_2SO_4$, 3.3 g NaCl, 20 g glucose |
| SCA - 0.05% | 1.7 g yeast nitrogen base without amino acids, 2 g drop out mix, 0.4% ethanol, 5 g $(NH_4)_2SO_4$, 3.3 g NaCl, 0.5g glucose |

**Supplementary Table 3:** Primers used in this paper.

| Gene Name | Forward | Reverse |
|---|---|---|
| ***CNAG_04956*** | ACGGTATCGTCTCCACCAAC | CCAAAGCCTCGGTAGGTACA |
| ***CNAG_00028*** | AACAATGCGGGAAGTGAATC | TTCCGCCTTATCCCTTTTCT |
| ***CNAG_03465*** | TTTGGGGCCCCTTAATTATC | GGATAGGTGCATGAGGAGGA |
| ***CNAG_04313*** | GGAGCGATTCATTGGTCATT | GTCGGTACGATCGTTGGAGT |
| ***CNAG_06909*** | GCCCATAGCACTAGGAGTCG | ATGTCATCCTGCGAATAGCC |
| ***CNAG_00003*** | GTGAAACTCGGAGCTTGGTC | TACAATGGGCTCCATGAACA |
| ***CNAG_04546*** | TTATCGCATGTCTCCCCTTC | GTGGAATAGGAATGGCATGG |
| ***ACT1*** | CCCACACTGTCCCCATTTAC | AACCACGCTCCATGAGAATC |
