# Supplementary material for: High-Throughput Yeast Aging Analysis for Cryptococcus (HYAAC) microfluidic device streamlines aging studies in Cryptococcus neoformans
Source: Commun Biol. 2019 Jul 10;2:256. doi: 10.1038/s42003-019-0504-5 (PMC6620289; doi:10.1038/s42003-019-0504-5)
Supplement: Supplementary file 2 — Description of Additional Supplementary Files [file 42003_2019_504_MOESM2_ESM.docx]

**Supplementary Movie 1:** A single *C. neoformans* cell trapped in a bucket, rolling as media flows over the cell.

**Supplementary Movie 2:** Time lapse of a single *C. neoformans* cell trapped in a bucket as daughter cells bud from the mother and are removed by the flow of media.

**Supplementary Data 1:** Source data for Figures 2 and 3 and Supplementary Figures 3 and 4.
