# Supplementary material for: High-Throughput Yeast Aging Analysis for Cryptococcus (HYAAC) microfluidic device streamlines aging studies in Cryptococcus neoformans
Source: Commun Biol. 2019 Jul 10;2:256. doi: 10.1038/s42003-019-0504-5 (PMC6620289; doi:10.1038/s42003-019-0504-5)
Supplement: Supplementary file 4 — Reporting Summary [file 42003_2019_504_MOESM4_ESM.pdf]

nature research

Corresponding author(s): Erika Orner, Bettina Fries

Last updated by author(s): Jun 3, 2019

Reporting Summary

Nature Research wishes to improve the reproducibility of the work that we publish. This form provides structure for consistency and transparency in reporting. For further information on Nature Research policies, see Authors & Referees and the Editorial Policy Checklist.

Statistics

For all statistical analyses, confirm that the following items are present in the figure legend, table legend, main text, or Methods section.

n/a	Confirmed	
☐	☒	The exact sample size (n) for each experimental group/condition, given as a discrete number and unit of measurement
☐	☒	A statement on whether measurements were taken from distinct samples or whether the same sample was measured repeatedly
☐	☒	The statistical test(s) used AND whether they are one- or two-sided<br>*Only common tests should be described solely by name; describe more complex techniques in the Methods section.*
☐	☒	A description of all covariates tested
☐	☒	A description of any assumptions or corrections, such as tests of normality and adjustment for multiple comparisons
☐	☒	A full description of the statistical parameters including central tendency (e.g. means) or other basic estimates (e.g. regression coefficient) AND variation (e.g. standard deviation) or associated estimates of uncertainty (e.g. confidence intervals)
☐	☒	For null hypothesis testing, the test statistic (e.g. F, t, r) with confidence intervals, effect sizes, degrees of freedom and P value noted<br>*Give P values as exact values whenever suitable.*
☒	☐	For Bayesian analysis, information on the choice of priors and Markov chain Monte Carlo settings
☒	☐	For hierarchical and complex designs, identification of the appropriate level for tests and full reporting of outcomes
☒	☐	Estimates of effect sizes (e.g. Cohen's d, Pearson's r), indicating how they were calculated

Our web collection on statistics for biologists contains articles on many of the points above.

Software and code

Policy information about availability of computer code

Data collection	PearlScope (EVOS software version 31384) was used in this study for data collection and is available through ThermoFisher.
Data analysis	Fiji software (v 2.0.0-rc-65/1.52b) and Prism 6 were used for data analysis during this study and are available online.

For manuscripts utilizing custom algorithms or software that are central to the research but not yet described in published literature, software must be made available to editors/reviewers. We strongly encourage code deposition in a community repository (e.g. GitHub). See the Nature Research guidelines for submitting code & software for further information.

Data

Policy information about availability of data

All manuscripts must include a data availability statement. This statement should provide the following information, where applicable:

- Accession codes, unique identifiers, or web links for publicly available datasets
- A list of figures that have associated raw data
- A description of any restrictions on data availability

Data generated or analyzed in this study that are not provided in the Supplementary Data are available through request from the corresponding author. AutoCAD design of the HYAAC device is available at figshare (10.6084/m9.figshare.8216267).

Field-specific reporting

Please select the one below that is the best fit for your research. If you are not sure, read the appropriate sections before making your selection.

☒ Life sciences ☐ Behavioural & social sciences ☐ Ecological, evolutionary & environmental sciences

Life sciences study design

All studies must disclose on these points even when the disclosure is negative.

Sample size	No sample size was pre-calculated. Samples sizes were all chosen based on sample sizes used in previous studies.
Data exclusions	The only data that was excluded were cells in the HYAAC device that did not stay in the device until the end of the RLS (i.e. if a cell divided 7 times and was lost before generation 8 was finished budding, that cell was not counted towards the strain's RLS)
Replication	All experiments were done in at least triplicates to ensure reproducibility. The only experiment that was not done in triplicate was the knock out susceptibility to AMB in the HYYAC at 0 and 10 generation. We felt that because we analyzed over 200 cells for each strain and because this was more of an initial screen, biological triplicates was not needed at this stage. Follow up studies with certain mutants pulled from this screen will be subjected to tests done in triplicate to confirm these findings and proceed forward.
Randomization	Randomization was not relevant to this study and no samples needed to be grouped into various categories.
Blinding	Blinding was not done for this study as it was not very relevant for data collection.

Reporting for specific materials, systems and methods

We require information from authors about some types of materials, experimental systems and methods used in many studies. Here, indicate whether each material, system or method listed is relevant to your study. If you are not sure if a list item applies to your research, read the appropriate section before selecting a response.

Materials & experimental systems

n/a	Involved in the study	
☒	☐	Antibodies
☒	☐	Eukaryotic cell lines
☒	☐	Palaeontology
☒	☐	Animals and other organisms
☒	☐	Human research participants
☒	☐	Clinical data

Methods

n/a	Involved in the study	
☒	☐	ChIP-seq
☒	☐	Flow cytometry
☒	☐	MRI-based neuroimaging
